# Supplementary material for: Comparative postoperative prognosis of ceramic-on-ceramic and ceramic-on-polyethylene for total hip arthroplasty: an updated systematic review and meta-analysis
Source: PeerJ. 2024 Sep 25;12:e18139. doi: 10.7717/peerj.18139 (PMC11438439; doi:10.7717/peerj.18139)
Supplement: Supplemental Information 2 [file peerj-12-18139-s002.docx]

Dear Editor,

On behalf of my co-authors, I am submitting the enclosed manuscript titled " Comparative Postoperative Prognosis of Patients Between Ceramic-on-Ceramic and Ceramic-on-Polyethylene for Total Hip Arthroplasty: An updated Evidence-Based Analysis" for consideration of publication in **PeerJ**. I understand the objectives of the journal and have formatted the manuscript in accordance with the style and needs of **PeerJ**. I also understand the procedure involved in the review process.

*In total hip arthroplasty, some people use ceramic head + ceramic lining, some people use ceramic head + polyethylene lining, which is better, has been controversial until now. Although three published meta-analyses compared postoperative complications and revision between the CoC-THA and CoP-THA groups,* ***neither paid attention to the evaluation of postoperative patient prognosis, nor did they assert which bearing surface was the best material for THA.*** *Whereafter, two novel original studies of the same topic have been published during 2021-2022. Thus, the purpose of this meta-analysis was to compare the results of the two bearing surfaces after THA objectively (complications and revision) and subjectively (Harris hip score), and to update the evidence, which could provide guidance for patients in preoperative selection of bearing surfaces.*

***To the best of our knowledge, previous articles on the selection of bearing surfaces for total hip replacement are all from the perspective of doctors, focusing on the outcome of the operation. However, this current report is the first to focus on postoperative outcomes from the patient's perspective and to provide an important reference for patients in preoperative selection of bearing surfaces for total hip replacement.***

We believe that our paper would arouse the interest of orthopedic surgeons. It has been edited for proper English language, grammar, punctuation, spelling, and overall style by highly qualified native English speaking professional editors at Textcheck.org. The English in this document has been checked by at least two professional editors, both native speakers of English. For a certificate, please see: http://www.textcheck.com/certificate/RaUFPX. Neither the research content nor the authors' intentions were altered in any way during the editing process. In addition, this manuscript has not been submitted, accepted or published elsewhere, nor is it under review by any other journals. All of the authors declare that we have no competing financial interests. We hope this manuscript meets the requirements for publication as a **Systematic review/Meta-analysis** in **PeerJ**, thus be available for commenting and debate by your readers, making our paper the start of a scientific conversation about material selection in total hip arthroplasty.

We deeply appreciate your consideration of our manuscript, and we look forward to receiving comments from the reviewers. If you have any questions, please don’t hesitate to contact me at the address below.

Best regards

Sincerely,

Tao Li M.D.

Department of Orthopaedics, The Affiliated Hospital of Qingdao University, Qingdao, 266003, China

Tel & Fax: +86-0532-82913558	E-mail: [qdult@qdu](mailto:qdult@qdu).edu.cn	59# Haier Road, Laoshan District, Qingdao, P.R.China, 100730
